# A healthy bladder: a consensus statement

**DOI:** 10.1111/j.1742-1241.2011.02763.x

**Published:** 2011-10

**Authors:** E S Lukacz, C Sampselle, M Gray, S MacDiarmid, M Rosenberg, P Ellsworth, M H Palmer

**Affiliations:** 1 University of CaliforniaSan Diego, San Diego, CA, USA; 2 University of MichiganAnn Arbor, MI, USA; 3 University of VirginiaCharlottesville, VA, USA; 4 Alliance Urology SpecialistsGreensboro, NC, USA; 5 Mid-Michigan Health CentersJackson, MI, USA; 6 University Urological Associates Inc. at Brown UniversityProvidence, RI, USA; 7 University of North Carolina at Chapel HillChapel Hill, NC, USA

## Abstract

A panel of experts in urology, urogynecology, nursing, and behavioral therapy convened in 2010 to discuss the importance of a healthy bladder on overall health. They determined that a consensus statement was necessary to raise awareness among the general public, healthcare providers, payors, and policymakers, with the goals of minimizing the impact of poor bladder health and stimulating primary prevention of bladder conditions. In this statement, ‘healthy’ bladder function is described, as well as internal and external factors that influence bladder health. It is suggested that primary prevention strategies should be aimed at providing education regarding normal lower urinary tract structures and functioning to the public, including patients and healthcare providers. This education may promote the achievement of optimal bladder health by increasing healthy bladder habits and behaviors, awareness of risk factors, healthcare seeking, and clinician engagement and reducing stigma and other barriers to treatment. Promoting optimal bladder health may reduce the personal, societal and economic impact of bladder conditions, including anxiety and depression and costs associated with conditions or diseases and their treatment. While adopting healthy bladder habits and behaviors and behaviors may improve or maintain bladder health, it is important to recognize that certain symptoms may indicate the presence of conditions that require medical attention; many bladder conditions are treatable with a range of options for most bladder conditions. Lastly, the authors propose clinical directives based on persuasive and convergent research to improve and maintain bladder health. The authors hope that this statement will lead to promotion and achievement of optimal bladder health, which may improve overall health and help minimize the effects of bladder conditions on the public, healthcare professionals, educators, employers, and payors. The advisors are in consensus regarding the recommendations for improving and maintaining bladder health presented herein.

**What's known**

An unhealthy bladder may be evidenced by the presence of symptoms or disease. Bladder conditions affect a large proportion of the world population, and are associated with substantial economic and humanistic costs to society.

**What's new**

A panel of experts in urology, urogynecology, nursing, and behavioral therapy determined that a consensus statement was necessary to raise awareness of the importance of a healthy bladder on overall health among the general public, healthcare providers, payors, and policymakers. It is suggested that primary prevention strategies should be aimed at providing education regarding normal lower urinary tract structures and functioning. Clinical directives are proposed to improve and maintain bladder health.

## Background

Bladder health is an important component of an individual's overall health. Recently, experts from a variety of disciplines convened to develop a bladder health initiative. This expert panel was convened and sponsored by Pfizer Inc and was composed of the seven authors and three additional individuals acknowledged at the end of this article. This panel determined that a consensus statement was needed to raise awareness of the importance of bladder health among the general public, clinicians, policy makers and public health officials. In this consensus statement, a definition for ‘bladder health’ and areas for intervention are proposed. The goal of this statement is to minimise the negative impact of poor bladder health on individuals who are directly or indirectly affected, such as patients, family members, healthcare professionals, educators, employers and payors, and to stimulate efforts that may lead to prevention of bladder conditions that affect overall well-being.

An unhealthy bladder may be characterised by the presence of one or more symptoms or disease such as increased voiding frequency or cancer. A number of conditions may cause lower urinary tract symptoms (LUTS), including but not limited to overactive bladder (OAB), bladder outlet obstruction (BOO), bladder pain syndrome/interstitial cystitis (BPS/IC), urinary tract infection (UTI) and bladder cancers (1). LUTS can be divided into three categories: storage, voiding and postmicturition (Table 1) (1). Storage symptoms include urinary incontinence (UI, including stress, urgency or mixed incontinence), increased daytime voiding frequency, nocturia and urgency. Voiding symptoms include hesitancy, slow stream, straining and spraying. Postmicturition symptoms include feeling of incomplete emptying and postmicturition leakage. BPS/IC is defined as an unpleasant sensation (pain, pressure discomfort) perceived to be related to the urinary bladder, associated with LUTS of more than 6 weeks duration, in the absence of infection or other identifiable causes (2)

**Table 1:** Classifications of LUTS

**Storage**	**Voiding**	**Postmicturition**
Urgency	Slow stream	Postmicturition dribble
Frequency	Splitting/spraying	Incomplete emptying
Nocturia	Intermittent stream	
Incontinence	Hesitancy	
	Straining	
	Terminal dribble	

### Prevalence of bladder conditions

Bladder conditions affect a large proportion of the world population. It has been estimated that in 2008 over 45% of the world population aged ≥ 20 years (1.9 billion people) were affected by LUTS (3). Approximately 8.2% (348 million) of the 2008 world population were estimated to be affected by UI, including 3.2% with stress UI, 1.2% with urgency UI, 1.3% with mixed UI and 2.5% with UI without symptoms of stress or urgency UI (3). The prevalence of OAB, defined by urgency, with or without urgency UI, usually with increased daytime frequency and nocturia, is approximately 12% (men, 11%; women, 13%) in Europe and North America (4), with almost 11% of the world population (455 million people) affected by OAB (3). The worldwide prevalence of LUTS suggestive of BOO in 2008 was estimated to be 21.5% (917 million people) (3). The prevalence of BPS/IC is difficult to determine because of the lack of validated questionnaires for use in epidemiological studies and misunderstanding of the definition of IC (5). However, one study conducted in the United States reported that the prevalence of BPS/IC symptoms among adults aged ≥ 30 years ranged from 0.83% to 2.71% in women and 0.25% to 1.22% in men (6), whereas another US study estimated that the prevalence among women aged ≥ 18 years was approximately 3–6% (7). UTI is among the most common reasons for treatment in adult primary care clinical practice (8). It is the most common infectious disease among women worldwide (excluding intestinal disease) and is associated with a high recurrence rate (9). Globally, bladder cancer is the 7th most common cancer in men and the 17th most common in women. However, the prevalence of bladder cancer varies among countries, with the highest prevalence in Western countries and lowest prevalence in Asian countries (10–12). The risk of bladder cancer and associated mortality increases with age (13).

### Financial and personal burden of bladder conditions

Bladder conditions are associated with substantial costs to society both economically and socially. The mean cost of routine care (incontinence pads, laundry, etc.) per person among women with UI in 2005 were estimated at approximately $492 ± 898 US dollars per year and increased with UI severity (14). In a study of women with stress UI, mean annual cost of incontinence management in 2007 was approximately $750, including the cost of pads and dry cleaning (15). Urinary incontinence is also associated with a high risk of nursing home placement, which bears significant financial and emotional burdens (16,17). The total (direct and indirect) costs associated with OAB in 2007 were estimated to be $65.9 billion in the US alone (18). Costs for BPS/IC are also substantial (19); annual per person costs in the USA in 2005 were estimated to range from $3631 (Medicare rates) to $7043 (non-Medicare rates) (20). There is also considerable annual cost associated with UTI, which was estimated to be approximately $1.6 billion in 2003 (21,22). Bladder cancer is associated with the highest lifetime cost per patient of any cancer, with the total annual cost estimated at $3.4 billion in the USA in 2002 (23).

In addition to the economic burden, bladder conditions negatively impact many aspects of the health-related quality of life (HRQL) (24). This is important, as the World Health Organization recognises that ‘health is not only the absence of infirmity and disease but also a state of physical, mental and social well-being’ (25). Emotional well-being, a component of overall health, is known to be affected by bladder health. For example, LUTS are associated with increased anxiety and depression, decreased physical activity, reduced work productivity (absenteeism) and impaired sexual function (26,27). OAB and UI are bothersome and both can be incapacitating conditions (26,28,29). UI is associated with depression in men and women, with prevalence of depression increasing with severity of incontinence (30–33). In men, UI is significantly associated with major depression (per 10-year increase, OR 2.7; 95% CI 1.6, 4.0) (30); similarly, depression is a predictor for urgency UI in women (32,34). UI may be associated with high-impact physical activity in young women, as high rates of UI have been reported in female athletes (35–38), dancers (38), fitness instructors (39) and women in the United States Military Academy (40). Among older men and women, OAB symptoms are associated with increased risk of falls and fractures, UTIs and perineal skin disorders including incontinence-associated dermatitis (41). BPS/IC is associated with lower HRQL, poor sleep and increased anxiety, stress and depression compared with controls (42).

The impact of UI on physical activity has far-reaching implications for prevention of cardiovascular disease, diabetes and other chronic illness. As UI severity increases, it imposes an increasingly greater barrier to physical exercise (43). Several types of bladder conditions, including OAB, UI, stress UI, LUTS and BPS/IC are associated with lost work productivity (26,27,44). Bladder cancer and its treatments also profoundly reduce HRQL in survivors and their families (45–48). The impact of poor bladder health on society is not fully appreciated by most healthcare professionals or the general public, including the affected individuals, which likely account at least in part for the underdiagnosis and undertreatment of many bladder disorders (49). We believe that by defining and promoting ‘bladder health,’ healthcare providers can improve the overall health of those directly and indirectly affected.

### Developing a bladder health consensus statement

The International Consultation on Incontinence (ICI) has provided guidance on promoting continence, including raising awareness among sufferers, educating healthcare providers and primary prevention of UI, as well as pelvic organ prolapse and faecal incontinence (50). Moreover, it has been demonstrated that bladder health programmes, such as community UI intervention and nurse continence programmes, can result in increased use of self-management strategies, reductions in UI episodes and incontinence pad usage, and improved HRQL (51–55). Our goals in issuing this statement are to discuss the importance of bladder health as a component of overall health and to describe factors that influence bladder health. The statement is directed towards the general public, clinicians, policy makers and public health officials, and we believe that increased education through a consensus statement addressing major issues related to bladder health may help improve overall bladder health.

We acknowledge that there are several challenges associated with developing a consensus statement regarding bladder health. It is difficult to define or describe ‘normal’ or ‘healthy’ bladder function, as it is not simply the absence of disease. We also acknowledge that the causal relationship between bladder conditions and concomitant conditions are not entirely understood, such as the relationship between urgency UI and depression. Healthy habits do not guarantee healthy, normal bladder function, and normal function can be present despite poor habits. In addition, there is a lack of research regarding healthy bladder habits and/or normal bladder function.

Many factors, both internal and external to the individual, may influence bladder functioning. However, providing educational materials regarding healthy habits and function to symptomatic individuals seeking care, individuals who have symptoms but are not seeking care, and asymptomatic individuals has been shown to improve health-seeking behaviours (51) and thus may be applicable to bladder health. Finally, it is unclear whether all conditions are preventable or whether it is only possible to delay onset or reduce bothersome symptoms. Limited research has been conducted for primary prevention (education about behaviour changes and setting expectations for normal urinary tract function) of bladder dysfunction; most studies are focused on secondary prevention (52,56).

## Discussion

### Understanding bladder anatomy

**Figure 1:**
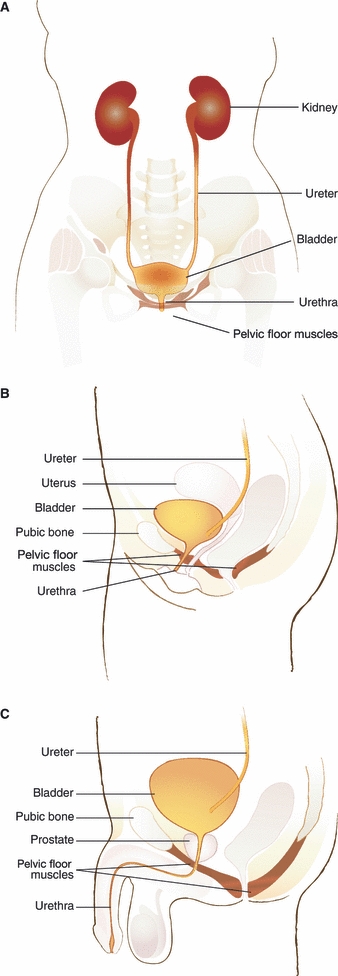
Urinary system (A) frontal view (woman), (B) lateral view (woman) and (C) lateral view (man)

Understanding the anatomy and physiology of the urinary system and what constitutes a healthy bladder, including its function, anatomy and potential susceptibility to dysfunction, may help promote bladder health. The bladder is a hollow muscular organ that is lined by mucosa (urothelium) and is sensitive to both urine volume and its chemical composition (Figure 1A–C). The kidneys filter blood to remove excess water and waste products and produce urine, which travel from the kidneys to the bladder through the ureters. Urine is stored in the bladder until it is emptied through the urethra. The bladder neck, external urethral sphincter and the pelvic floor muscles help to maintain continence.

### Healthy bladder functioning

A healthy bladder is free of bacterial infection or tumours and stores urine without discomfort at low pressure with intermittent signals of filling (57). Normal functional bladder capacity in adults ranges from approximately 300 to 400 ml (58,59). Although the International Continence Society defines urinary frequency as the perception by the patient that he/she voids too often (1), epidemiological studies suggest that the normal micturition rate is approximately 8 micturitions per day and 1 or fewer episodes per night (60). As such, small volumes with increased micturition frequency suggest abnormality. Nerve fibres respond to increasing filling, and micturition is prompted at or near bladder volume capacity (approximately every 3–4 h based on volume of liquid consumed). Voiding typically occurs via initial relaxation of the pelvic floor muscles and the bladder neck followed by voluntary contraction of the detrusor muscle. Healthy voiding occurs promptly with strong continuous flow and complete emptying without pain or blood in the urine. When necessary, an individual should be able to defer voiding without leakage. Variations in any of these normal responses may be a sign of disease.

Knowledge of the physical and functional aspects of the bladder may help promote bladder health, as well as treatment outcomes for bladder conditions. For example, pelvic floor muscle exercises (also known as Kegel exercises) may prevent and treat mild stress incontinence (61–63). Many individuals with OAB who were successfully treated with a combination of behavioural interventions plus an antimuscarinic after failing previous antimuscarinic therapy cited information provided to them about the bladder and pelvic floor muscles as an important contributor to their treatment satisfaction (64). It is anticipated that increasing knowledge about changes in bladder health/function over time will help adults to identify and seek treatment for bladder conditions. Anatomically, functional bladder capacity increases with age from childhood [(years of age + 2) × 30 ml] to adulthood (300–400 ml). Changes in the adult bladder and pelvic floor muscles with ageing also include decreased bladder sensation, decreased contractility during voiding, decreased muscle tone in pelvic floor muscles and increased residual volume (57,65–70). Other physical changes include up-regulation of purinergic receptors with increased prevalence of detrusor overactivity (71) and increased acetylcholine release in the urothelium (71), both of which can produce LUTS. The prevalence of LUTS increases with age (4). While there are age-associated changes to the bladder and its function, those effects are minimal and easily compensated for by changes in bladder habits. Troublesome or severe bladder symptoms are not normal; therefore, the false belief that incontinence, for example, is a natural, inevitable consequence of ageing should be dispelled (72).

### Influences on bladder function

#### Dietary influences

**Figure 2:**
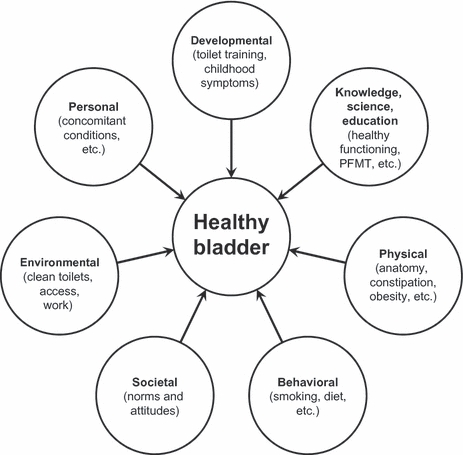
Factors influencing bladder health

A number of factors influence bladder function (Figure 2). For example, **s**ome foods and beverages may provoke bladder urgency or discomfort or stimulate diuresis, including caffeine, carbonated beverages and artificial sweeteners (73–77). Several types of beverages and foods, including coffee, tea, soda, alcoholic beverages, citrus fruits and juices, artificial sweeteners and hot peppers can exacerbate symptoms in patients with BPS/IC (78). The BPS/IC Ad Hoc Committee on Diet has published a list of ‘bladder- and prostate-friendly foods’ with the intent of helping people determine and avoid foods that may trigger symptoms or compromise treatment efficacy (79) and AUA BPS/IC guidelines suggest avoidance of foods and beverages which are known to be bladder irritants, such as coffee and citrus products. The guideline also suggests using an elimination strategy to identify foods that may cause or exacerbate symptoms (2). Fluid intake may also affect bladder function. Based on 3-day diary entries, fluid intake averaging > 3700 ml per day has been associated with voiding frequency of > 10 times during the day and nearly 2 times at night, as well as higher incidence of UI, compared with intake of approximately 2400 ml per day (80). Some people may limit fluid intake as a way to cope with LUTS, including urgency, frequency and UI (81–83). However, fluid restriction may increase urine concentration, leading to irritation of the bladder mucosa and increased incidence of LUTS and UTI (84). The US Food Science Board recommended that fluid intake volume is 30 ml/kg per day or half an ounce per pound per 24 h.

#### Pelvic floor functioning

Healthy pelvic floor function is important to having a healthy bladder. Pelvic floor muscle training can be used to alleviate UI and urgency and to extend the interval between voids (85). Strengthening or learning to contract pelvic floor muscles may also increase the efficacy of pharmacotherapy (85,86). In a randomised clinical trial, pelvic floor muscle training, pre-emptive pelvic floor contraction to prevent leakage and bladder training significantly decreased UI and voiding frequency 12 months postintervention (87). In another study, education about pre-emptive pelvic floor muscle contraction to prevent leakage (the Knack maneuver) significantly reduced UI in older women and pregnant women (88). A systematic review assessing the effect of pelvic floor muscle training versus standard antenatal and postnatal care concluded that pelvic floor muscle training may prevent UI in late pregnancy and postpartum and may effectively reduce persistent postpartum UI (89). Timed voiding has also been employed with and without other behavioural interventions for managing UI (90). A test of education about bladder training, pelvic floor muscle training and the Knack maneuver is currently in progress to assess the preventive impact of these combined behavioural interventions.

#### Adult and childhood toileting habits

Toileting behaviours related to urination can contribute to dysfunctional voiding habits. Identifying and modifying the various components of voiding, including place, time, position and style, may help prevent, eliminate, reduce and/or manage LUTS (91). Furthermore, toilet training rituals during childhood may affect bladder health in adulthood (92) and childhood urinary symptoms are significantly associated with adult bladder conditions (93,94). An association has also been reported between OAB symptoms in children and their birth parents, with the fathers having stopped ‘bed wetting’ at a significantly later age than the fathers of children without OAB and mothers of children with OAB having similar symptoms in childhood compared with mothers of children without OAB (95).

#### Societal attitudes

Social/cultural attitudes may influence the response to or perception of bladder function and may interfere with treatment-seeking behaviour. LUTS such as increased voiding frequency and urgency are associated with social limitation, loss of control of the body and speculation as to the nature of a non-specific ‘problem’ (96). UI and other LUTS are associated with reduced HRQL, stigma and embarrassment (28,96–98). Bladder conditions may interfere with cultural/religious rituals, resulting in underreporting. For example, Jewish and Muslim religions require an element of cleanliness for prayer that is compromised by the perceived lack of cleanliness associated with urinary incontinence (99).

#### Environmental influences

Environmental factors can also influence bladder health. Work environments are required to provide adequate access, cleanliness and safety for toilet facilities (100); lack thereof can cause decreases in liquid intake (101) or infrequent voiding (102) by workers that can lead to compromised bladder function or urinary tract infection. Occupational barriers exist for individuals with paruresis (103), who experience social anxiety associated with travel, work or interpersonal relationships associated with the fear of being unable to urinate in the presence of others. In addition, attitudes and rules regarding toileting in school may influence bladder habits. Teachers’ awareness of factors that contribute to bladder dysfunction in school-aged children is important (104,105). Lack of awareness among teachers regarding normal elimination patterns in children can lead to restrictions on the frequency of children's access to bathroom facilities, which may have adverse effects on bladder health (104). In addition, the bathrooms in schools are reportedly often lacking in privacy, cleanliness and safety (e.g. a place where bullying may occur), which may contribute to urine-holding behaviours and dysfunctional voiding (104,106).

#### Physical factors

Physical influences on bladder health are less understood. Bladder conditions can vary based on age, gender, health history (107,108) and ethnicity (109–111). For instance, it is well established that the prevalence of all categories of LUTS increase with age in men and women (4,112). Moreover, there are differences between men and women in the LUTS that they are likely to experience and in the bother associated with LUTS (113–116). In the EPIC study, a population-based, cross-sectional survey of UI, OAB and other LUTS among people in five countries, the prevalence of storage LUTS tended to be greater in women (59% vs. 51%), whereas the prevalence of voiding (26% vs. 20%) and postmicturition (17% vs. 14%) LUTS tended to be higher in men (4). Men may be more likely to experience bother associated with UI than women (117).

UTI is more prevalent in women than men (118) and a variety of factors affecting UTI in women may be related to age. For example, in women, postmenopausal status, sexual activity, history of UTI, treated diabetes and UI are associated with a higher risk of UTI (119,120). UTI in postmenopausal women has been linked to several factors/conditions that are associated with a decrease in oestrogen, including vaginal prolapse, elevated postvoid residual urine volume and urinary incontinence. Treatment with oestrogen results in increased glycogen production in the vaginal tissues providing an acidic environment for lactobacilli and other normal pathogens to thrive. Low oestrogen levels result in alterations in pH and promotion of growth of enteric bacteria associated with UTI. Treatment with vaginal oestrogen vs. placebo has shown promise for utilisation of oestrogen for reducing the incidence of recurrent UTI (121). At clinical presentation, postmenopausal women are more likely than younger women to report flank pain, whereas younger women are more likely than postmenopausal women to report frequency, dysuria, haematuria and fever (122). BPS/IC may also be more prevalent in women than in men (123). Bladder cancer is more prevalent in men than in women and the risk of bladder cancer increases with age in both genders (13).

#### Concomitant conditions

Bladder health may also be influenced by secondary effects of pregnancy (124) and childbirth (124–127) and by a number of conditions or comorbidities, such as obesity (128,129), diabetes (130), hypertension/heart failure (101,130) and constipation. Furthermore, patients may have comorbidities that are not only reportedly associated with increased rates of incontinence but increase the difficulty of managing UI symptoms (131). For example, dementia has been shown to be an independent predictor of UI (132). In addition, many medications prescribed for comorbidities (e.g. sympathomimetics, tricyclic antidepressants, α-adrenergic blockers and angiotensin converting enzyme inhibitors) may affect continence (133). Although urinary incontinence is prevalent in patients with heart failure and/or comorbid diabetes, patients vary with regard to health-seeking behaviours to address urinary incontinence thereby requiring different types/levels of education (130). Obesity is associated with LUTS, including stress, mixed and urgency UI (128,134,135). Likewise, weight loss is associated with improvements in incontinence status (128,136). Although the exact mechanisms linking the two conditions has not been completely elucidated, results of the Program to Reduce Incontinence by Diet and Exercise (PRIDE) study indicate an association between BMI and abdominal circumference with intra-abdominal and intravesical pressure (137). Shared neural pathways for the bladder and bowel may play a role in OAB symptoms (138,139); individuals with constipation are more likely to develop OAB symptoms than those who are not constipated (140). Furthermore, constipation and OAB are associated with uterovaginal prolapse; in a study of 320 women with LUTS (40% with OAB), 16% had faecal incontinence (FI) and 32% had constipation (141). Finally, bladder cancer is more common in individuals with a history of smoking, exposure to aniline dyes, history of chronic bladder inflammation, prior pelvic irradiation (such as for prostate cancer or gynaecological malignancies), chemotherapy with cyclophosphamide (cytoxan) or ifosfamide (ifex) and in those with chronically low fluid consumption.

Conversely, there may be collateral physical benefits of enhanced bladder health. The increased risk of falls and fractures in patients with OAB or urgency UI is thought to be largely attributable to the act of rushing to the bathroom. Thus, treatment of OAB and UI may be associated with decreased fall risk. (35,142,143). Additional benefits of healthy bladder habits may include improved sexual function. Enhanced sexual pleasure is most plausibly linked to greater pelvic muscle strength and control ensuing from an exercise regimen recommended to reduce UI. Research is sparse, but results of two studies provide some support. Midlife women with stress UI who completed a 6-month programme of pelvic floor muscle training reported fewer problems with sexual dysfunction related to UI (144). Midlife women who completed a 12-month programme of pelvic floor muscle training reported significantly higher levels of sexual satisfaction compared with controls (145).

An important modifiable behaviour associated with poor bladder health is smoking. There are strong associations between smoking and LUTS (146–149) and the risk of bladder cancer has been reported to be increased twofold in smokers with an increase in risk with increasing amounts of smoking (150) and longer duration (151). In addition, smoking cessation has positive impact on overall health and related quality of life and should universally be encouraged.

### Potential benefits of the consensus statement on bladder health

This statement is intended to raise awareness of bladder health by stimulating discussion on this topic and primary prevention research in the form of epidemiological studies, educational needs assessment and community-based public health initiatives. Primary preventions are intended to prevent expected health problems, to maintain existing states of health and healthy functioning and to promote desired outcomes (56,152). Thus, initiatives that disseminate information about healthy bladder function may give rise to adoption of practices that promote bladder health.

The desired outcome of increased awareness of healthy bladder habits is a reduction in negative outcomes associated with bladder conditions including nursing home admittance, falls and impaired HRQL (17,35,142,143). Individuals with healthy habits may still develop bladder conditions; however, knowledge of the physical and functional aspects of the bladder may help mitigate the sequelae of underreported, underdiagnosed and undertreated bladder symptoms. Identification and awareness of risk factors, including smoking and obesity, is likely to be an important part of primary prevention of bladder conditions. In addition, bladder health education may increase screening for bladder cancer, BPS/IC, early and transient UI and UTIs, for which some diagnoses are ambiguous. For BPS/IC in particular, there remains a lack of consensus on diagnosis based on the lack of a diagnostic instrument to accurately diagnose the condition, which may be attributable at least in part to the overlap in symptomatology, including pelvic pain and UI, with various other conditions (e.g. fibromyalgia, endometriosis, irritable bowel syndrome and recurrent UTIs) (5). Education, however, should improve outcomes and/or perceptions about bladder health. A study evaluating the effect of an education intervention, including information about OAB, medication use and behavioural therapy, demonstrated that adherence to behaviour modification therapies and self-perception of treatment outcome were significantly enhanced compared with controls (153). Adherence with a self-management practice such as pelvic floor muscle training has been associated with adherence to bladder training as well (154).

Many people with bladder conditions tend not to report related symptoms to healthcare providers and use multiple coping mechanisms as opposed to seeking professional treatment (155). Various health risks are associated with avoidance of healthcare seeking, including UI progression and recurrence of UTI (155). Men and women vary with regard to perception of bother and healthcare-seeking behaviours (113). Raising awareness can help reduce the stigma associated with bladder conditions, which is a barrier to bladder health that can compromise outcomes and reduce HRQL (156). Improving the basic understanding of normal bladder function and treatment options for conditions/disease may result in increased care-seeking behaviours. Education can help reduce the stigma associated with LUTS (96). Increasing physician, nurse practitioner and physician assistant education about the importance of bladder health may also increase clinician engagement by combating various barriers to clinician involvement (e.g. limited reimbursement for prevention, lack of clinician training, etc.).

An anticipated benefit from this consensus statement is to raise awareness among the Public Health world regarding bladder health in the context of overall health, to help lay the ground work for a world-wide public health initiative (157). One limitation of this initial statement is that it is based on the US perspective, because, globally, the impact of the influencers will vary. In addition, the authors of this statement acknowledge gaps in the available literature. Lastly, the authors of this statement do not intend it to be a comprehensive review of all data related to bladder health.

## Summary

Bladder health is a key component of overall health. Internal and external factors influence bladder health. Primary prevention through education regarding normal bladder structures and functioning can help promote healthy bladder habits and early treatment seeking for bladder conditions. Our goal is to raise awareness about bladder health that, in turn, will reduce the associated personal, societal and economic burden, including anxiety and depression related to stigma and costs associated with bladder conditions. Increases in healthcare-seeking behaviours should result from knowledge that most bladder conditions are treatable and a range of treatment options exist, including self-management.

Promoting and achieving optimal bladder health can help minimise the effects of bladder conditions on the affected population, healthcare professionals, educators, employers and payors. Previous bladder health initiatives have focused largely on primary prevention of incontinence, often exclusively in women. This is the first US public health statement to address bladder health and is intended to promote wide-spread bladder health awareness in the context of overall health. Consensus was reached that there are established clinical directives that are generally agreed upon to promote and maintain bladder health. These include: (i) consume an adequate amount of fluid (25–30 ml/kg per day, the amount needed to empty the bladder every 3 to 4 h), (ii) moderate consumption of foods or beverages known to irritate the bladder, (iii) adopt a relaxed position for urination and allow time for the bladder to empty, (iv) use self-management practices of pelvic floor muscle training, bladder training and pre-emptive pelvic floor contraction to improve and maintain bladder health, (v) avoid constipation, (vi) avoid obesity; and (vii) do not smoke.
